# Breakthroughs in the discovery and use of different peroxidase isoforms of microbial origin

**DOI:** 10.3934/microbiol.2020020

**Published:** 2020-09-22

**Authors:** Pontsho Patricia Twala, Alfred Mitema, Cindy Baburam, Naser Aliye Feto

**Affiliations:** OMICS Research Group, Department of Biotechnology, Vaal University of Technology, Vanderbijlpark, South Africa

**Keywords:** microbial peroxidases, peroxidase isoforms, enzymes, peroxidases, degradation, pollutants

## Abstract

Peroxidases are classified as oxidoreductases and are the second largest class of enzymes applied in biotechnological processes. These enzymes are used to catalyze various oxidative reactions using hydrogen peroxide and other substrates as electron donors. They are isolated from various sources such as plants, animals and microbes. Peroxidase enzymes have versatile applications in bioenergy, bioremediation, dye decolorization, humic acid degradation, paper and pulp, and textile industries. Besides, peroxidases from different sources have unique abilities to degrade a broad range of environmental pollutants such as petroleum hydrocarbons, dioxins, industrial dye effluents, herbicides and pesticides. Ironically, unlike most biological catalysts, the function of peroxidases varies according to their source. For instance, manganese peroxidase (MnP) of fungal origin is widely used for depolymerization and demethylation of lignin and bleaching of pulp. While, horseradish peroxidase of plant origin is used for removal of phenols and aromatic amines from waste waters. Microbial enzymes are believed to be more stable than enzymes of plant or animal origin. Thus, making microbially-derived peroxidases a well-sought-after biocatalysts for versatile industrial and environmental applications. Therefore, the current review article highlights on the recent breakthroughs in the discovery and use of peroxidase isoforms of microbial origin at a possible depth.

## 1. Introduction

Enzymes are biological catalysts that facilitate the conversion of substrates into products providing favorable conditions that lower the activation energy of the reaction [1]. They play an important role as cost-effective and environmentally sensitive substituents for chemical processes in industries such as textile manufacturing, pharmaceutical, laundry, pulp and paper production [2],[3]. They are derived from different sources such as plants, animals and microorganisms [2],[4],[5].

Peroxidases are ubiquitous enzymes belonging to the class of oxidoreductase [4],[6]–[14]. They generally catalyze a variety of oxygen-transfer reactions between hydrogen peroxide or other peroxides as electron acceptors and many kinds of substrates (xenobiotics, lignin and other phenolic compounds) by means of oxygen (O_2_) liberation from H_2_O_2_ [6],[14].

The reduction of peroxides at the expense of electron donating substrates is what makes peroxidase enzymes useful in several biotechnological applications. Many peroxidase enzymes of particular interest are already being used as organic catalysts in numerous processes in various industries (Table 1) [8],[11],[19],[20].

**Table 1.** Peroxidase isoforms, their sources and industrial applications.

Enzyme	Substrate	Source	Application	Reference
Manganese peroxidase (MnP)	Lignin and other phenolic compounds	*Rigidoporous Lignosus*, *Ceriporiopsis subvermispora, Bacillus anthracis, Bacillus cereus*	Paper and pulp industry, textile industry, food industry, plastic degradation	[1],[19]–[22]
Lignin peroxidase (LiP)	Halogenated phenolic compounds, polycyclic aromatic compounds	*P. chrysosporium*, *Chrysonilia sitophila*, *Streptomyces* sp., *Bacillus cereus*	Textile industry, paper and pulp industry, plastic degradation, bioremediation, biopulping	[1],[19],[21]–[23]
Versatile peroxidase (VP)	Methoxybenzenes and phenolic aromatic compounds	*P. chrysosporium*, *Trametes versicolor*, *Pleurotus ostreatus*	Industrial biocatalyst, bioremediation	[1],[21],[24]
Glutathione peroxidase (GPx)	Lipid hydroperoxides, hydrogen peroxide	Grass carp, silver carp Southern bluefin tuna	Lignin degradation, dye decolorization, bioremediation	[8],[25],[26]
Dye-decolorizing peroxidase (DyP)	Phenols, hydroquinone, dyes, amines, aromatic alcohols and xenobiotic	*Bacillus subtilis*, *Phanerochaete chrysosporium*	Dye degradation, phenol degradation	[8],[27],[28]

Lignin peroxidase (LiP) and MnP are both heme proteins, used in the hydrolysis of lignocellulosic agricultural residues for the degradation of complex and recalcitrant constituent lignin. They are also used in bio-remediation, stabilization of wine and juices, denim washing, pollution control and in the treatment of industrial effluents and other xenobiotics [4],[15],[29]–[32].

Peroxidases are widely distributed in plants, animals and microorganisms, where they protect the cells against the effects of oxidative stress and cell damage due to H_2_O_2_ [3],[6],[8],[15]–[18]. Bacteria and fungi are the most frequently used microorganisms for industrial enzyme production. This is due to the fact that microbial sources are cheaper, their enzyme production and secretion systems are well-know and more controlled, hence they are preferred over other enzyme sources such as animal or plant [33]. However, the ability of an enzyme to be applied in various industrial areas involve the capacity of the enzyme to work under mild conditions at a high reaction rate, the ability to function under a wide range of pHs, at high temperature and pressures, of which in most cases depends on the enzymes origin (the producer).

*Bacillus* spp. are among the most frequently used enzyme producing bacteria for a number of reasons. *Bacillus* produces over a dozen of biologically active molecules and different types of enzymes, making it a high potential for biotechnological and biopharmaceutical applications [33]. *Bacillus* spp. are difficult to eliminate from materials and frequently cause contamination, but on the other hand such resistance can be of great advantage in biotechnological processes, especially those that require the use of microbes that can withstand harsh conditions such as extreme temperature.

The ability of different *Bacillus* species to ferment in the acid, neutral, and alkaline pH range, combined with the presence of thermophiles in the genus has led to the development of a variety of new commercial enzyme products with the desired temperature, pH activity and stability properties to address a variety of specific applications. Among the *Bacillus* species, *Bacillus subtilis* is the most frequently studied. Because of its ready genetic manipulation *B. subtilis* has become the model agent in laboratory research and its adaptability to changing environmental conditions has led to its success in industrial production [33].

## 2. Peroxidase enzymes

Peroxidases (EC number 1.11.1.x) represents a large family of oxidoreductases known to catalyze the oxidation of a variety of inorganic and organic substrates using H_2_O_2_ [16],[21]. They have a common catalytic mechanism for the degradation of H_2_O_2_ which is a two-electron oxidation-reduction with three distinct steps (section 2.2).

These enzymes are widely distributed in nature and are found in both eukaryotic and prokaryotic organisms [16]. They are involved in biological processes such as the breakdown of toxins, heavy metal detoxification and hormone regulation [6]–[9],[13],[16]. Additionally, they are used as catalysts in various industries, in clinical biochemistry, enzyme immunoassays, and removal of peroxides from industrial wastes and synthesis of various aromatic chemicals [3],[6],[9],[13],[21],[34].

### 2.1. Classification of peroxidase enzymes

**Figure 1.**
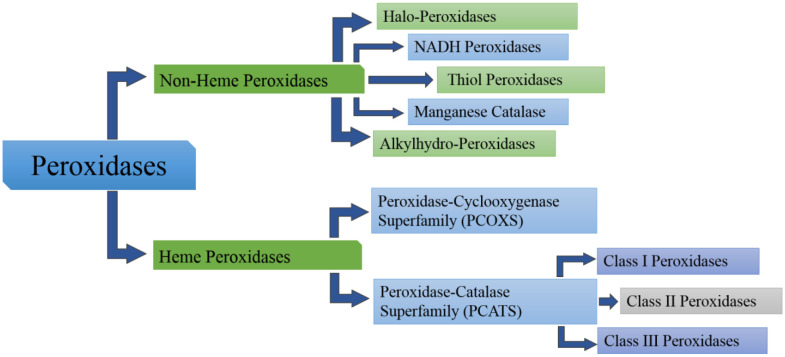
A schematic representation illustrating the classification of peroxidases.

Peroxidases are classified based on the presence or absence of a heme group [21]. The heme peroxidases stipulates approximately 80% of known peroxidases while the heme-free peroxidases cover the remaining 20% according to PeroxiBase database [21]. The heme group is further sub-divided into two major super families; peroxidase-cyclooxygenase superfamily (PCOXS) and the peroxidase-catalase superfamily (PCATS) (Figure 1) [8],[13],[21],[34].

**PCOXS:** The PCOX superfamily previously known as the animal peroxidase superfamily is composed of animal peroxidases such as myeloperoxidase (MPO), lacto-peroxidase (LPO) and thyroid peroxidase (TPO), suggested to be involved in innate immunity and defense response. The prosthetic heme group of the PCOX superfamily is covalently linked with the Apo-protein [21].

**PCAT:** The PCAT superfamily previously known as the plant, fungal and bacterial heme peroxidase group is one of the most studied super families of the non-animal heme peroxidases.

These superfamily includes plant peroxidases along with heme-containing peroxidases from fungi and bacteria, and it is further sub-divided into three classes (Figure 1) based on origin, amino acid homology and metal-binding capability [21].

- **Class I peroxidases:** These includes prokaryotic and eukaryotic intracellular peroxidases of non-animal sources, such as cytochrome *c* peroxidase (CCP), ascorbate peroxidase (APX) and catalase-peroxidase (CP). This class of peroxidase play significant role in the prevention of oxidative stress in bacteria as a result of their unique catalytic capability to dismutate H_2_O_2_ and their ability to evolve molecular O_2_ [21],[35].
- **Class II peroxidases:** These are exclusively fungal peroxidases. This class plays a major role in lignin biodegradation. It includes the white-rot fungal LiP, MnP and versatile peroxidases (VP) [21].
- **Class III peroxidases:** Are widely distributed in the plant kingdom consisting mainly of extracellular plant peroxidases such as horseradish peroxidase (HRP), soybean peroxidase (SBP) [21],[35] and Turpin peroxidase (TP) [35],[36]. This class of peroxidases is involved in a wide range of physiological processes such as cell wall metabolism, lignification, wound healing, auxins metabolism and defense against pathogens [17],[21],[34]. Unlike class I peroxidases which lack the disulfide bridges, calcium and an endoplasmic reticulum signal sequence at the structural level, class II & class III peroxidases both contain N-terminal signal peptides, four conserved disulfide bridges (differently located for each class) and calcium in their structure [21].

The non-heme peroxidases are not evolutionarily linked and form five independent families namely thiol peroxidases, alkylhydro-peroxidase, non-heme halo-peroxidase, manganese catalase and NADH peroxidase. Among all these thiol peroxidase family is the largest having two subfamilies namely glutathione peroxidases and peroxy redoxins [1],[21].

### 2.2. Mechanism of action of peroxidase enzymes

Plants and animals use peroxidases for quick and efficient decomposition of toxic peroxides [3]. Peroxidases are part of the antioxidative protection system [3] and are involved in the breakdown of a variety of substrates (ferricyanides and ascorbate) into harmless components through the transfer of O_2_ from H_2_O_2_ or other peroxides [6],[13],[16],[21],[37].

Peroxidases catalytic cycle is a three-step process involving distinct intermediate enzyme forms:

$$\begin{array}{c} \text{Step }\left(\text{i}\right).\text{ Peroxidase}+{\text{H}}_{\text{2}}{\text{O}}_{\text{2}}\to \text{Compound I}+{\text{H}}_{\text{2}}\text{O}\\ \text{Step }\left(\text{ii}\right).\text{ Compound I}+\text{PS}\to \text{Compound II}+\text{FRP}\\ \text{Step }\left(\text{iii}\right).\text{ Compound II}+\text{PS}\to \text{Peroxidase}+\text{FRP}+{\text{H}}_{\text{2}}{\text{O}}_{\text{2}}\\ \text{2 Substrate }\left(\text{reduced}\right)+{\text{H}}_{\text{2}}{\text{O}}_{\text{2}}\to \text{2 Product}\left(\text{oxidized}\right)+{\text{2 H}}_{\text{2}}\text{O} \end{array}$$

*PS: peroxidase substrate, FRP: free-radical product.

In the initial stages, the native ferric enzymes are oxidized by H_2_O_2_ resulting in an unstable intermediate, Compound I. Compound I contain a heme structure of Fe IV = O-porphyrin *π*-cation radical with consequent reduction of peroxide to water. In the second step, Compound I oxidizes an electron donor substrate (PS) to give rise to Compound II thereafter releasing a free radical product (FRP). Compound II is then further reduced by a second substrate molecule in step three regenerating the iron (III) state and producing another FRP [13],[21],[37].

## 3. Sources of peroxidase enzymes

Peroxidases are diversely distributed in nature and produced by array of sources including microbes, animals and plants [21]. Their distinctive features are based on the source and the subsequent sections describes in detail the major sources.

### 3.1. Plant-derived peroxidases

Plant peroxidases (class III) are a large multigene family in plants, primarily found in the cell wall and vacuoles. They are involved in a broad range of physiological processes such as lignin and suberin formation, auxin metabolism, cell elongation, plant defense, wound healing, as well as the generation of highly reactive oxygen species (ROS) [4],[17],[21],[34],[38]. They are also involved in air pollution damage control, bio-bleaching process, soil and water detoxification where they catalyze the conversion reaction of H_2_O_2_ to H_2_O [34],[39].

Plant peroxidases are used in a variety of biotechnological applications [8],[11]. Even though, their exploitation is often hampered by their notoriously difficult heterologous expression and limited stability, plant peroxidases are rendered as attractive biocatalysts due to their neutral optimum pH, their wide substrate specificity which enables them to use various synthetic chromophores as electron donors [40].

Thousands of peroxidase plant sources have been studied in the past [41]. Some of the major sources of peroxidases such as manganese peroxidase, lignin peroxidase and horseradish peroxidase are from papaya (*Carica papaya*), bare (*Acorus calamus*) and banana (*Musa paradisiacal*) [6],[9],[14],[34]. Horseradish peroxidase (HRP) isoenzymes are the most widely studied enzymes due to their wide specificity for H_2_ donors, stability, broad substrate specificity, superior catalytic efficiencies, tolerance to wide pH and temperature range [10],[13],[16],[37],[42]. Additionally, these enzymes are extensively used in the synthesis of various aromatic chemicals, diagnostic kits, ELISA, and removal of peroxides from industrial wastes [6],[34].

### 3.2. Microbial derived peroxidases

Microbially produced peroxidases from bacteria (*Bacillus subtilis, Bacillus sphaericus*, *Escherichia coli*, *Citrobacter* spp., *Pseudomonas* spp.), Cyanobacteria (*Anabaena* spp.), fungi (*Coprinopsi cinerea*, *Candida krusei*, *Phanerochaete chrysporium*), actinomycetes (*Thermobifida fusca, Streptomyces* spp.) and yeast have been extensively explored through various studies [6],[13]. They are widely used in the production of animal feedstock, decomposition of pollutants, sewage treatment, dye decolorization and lignin degradation in paper-pulp industries [6],[13].

The broad biochemical diversity and susceptibility to genetic manipulation render microorganisms an excellent source of enzymes. Studies have shown that peroxidase enzymes of microbial origin are more stable than those of plant or animal origin due their ability to survive the elevated temperature, high or low alkalinity and salt concentration conditions used in industrial applications processes [13].

*Bacillus* spp. have the ability to replicate rapidly and are resistant to extreme environmental conditions due to spore formation. Under nutrient-limiting conditions, *Bacillus* spp. differentiates into spores, which are metabolically dominant cells capable of surviving environmental stress, nutrient deficiency and extreme temperature [43]. Microbial enzyme technology has many advantages due to enzyme efficiency, sustainability, and low-cost effectiveness.

### 3.3. Animal-derived peroxidases

Animal peroxidases belong to the PCOXS superfamily and they are responsible for biological processes such as innate immunity, defense responses, immune system or hormone regulation [1],[11],[21]. Enzymes such as myeloperoxidase (MPO), lactoperoxidase (LPO) and thyroid peroxidase (TPO) belong to this group of peroxidases. The prosthetic heme group of these peroxidases is covalently linked with the apo-protein and they have been extracted from sources such as the southern bluefin tuna (*Thunnus maccoyi*), the grass carp (*Ctenopharyngodon idellus*) and Antarctic fish (*Trematomus bernacchi*) [8],[21],[25]. Their application is often hampered by low temperature stability, sensitivity to salt and organic solvents [10],[13].

## 4. Peroxidase isoforms

Various peroxidase isoforms have been discovered throughout the years, each having their own distinct features such as novel substrate specificity and distinct protein folding. Most peroxidases have significant roles they play both in nature and in various industrial applications

### 4.1. Glutathione peroxidases (GPxs)

Glutathione peroxidases (GPxs) (EC 1.11.1.9) are vital antioxidant enzymes which use glutathione or thioredoxins as an electron donor to catalyze the reduction of hydroperoxides to alcohols along with the oxidation of thiols to disulfides [25],[44].

**Figure 2.**
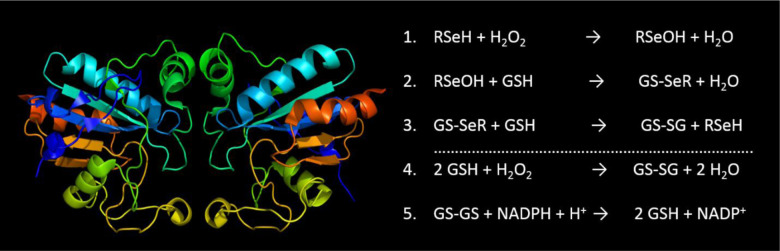
3D structure and general mechanism reaction of glutathione peroxidase [25],[44].

Figure 2 illustrates the 3D structure of GPx1 and the general mechanism reaction of peroxidase enzymes.

In the initial stage (Figure 2, reaction 1) the selenol of a selenocysteine reside is oxidized by H_2_O_2_ yielding a selenenic acid group (RSeOH). Selenenic acid group is then converted back into the selenol by a two-step process, first one being the reaction of GSH (reduced monomeric glutathione) to form GS-SeR and water (reaction 2), secondly the reduction of GS-SeR (reaction 3) intermediate to senenol by a second GSH molecule which then results in the release of GS-SG (glutathione disulfide) as a by-product. Finally, the glutathione peroxidase thereafter reduces the oxidized glutathione to complete the cycle (reaction 5) [25].

Glutathione peroxidases are widely distributed in oxybiotic organisms where they maintain intra-organic oxidative status balance by catalyzing the reduction of various hydroperoxides and H_2_O_2_ into corresponding alcohols and water [25]. In the mammal family, there are eight GPx isoforms (GPx1–GPx8) which are divided based on their cellular location, structure and specific substrate [25],[44]. GPx1, GPx4, and GPx6 are selenoproteins which contain the unique amino acid selenocysteine (Sec) while the remaining three (GPx5, GPx7 & GPx8) have cysteine instead of Sec hence they are non-selenoproteins [25].

GPx1 is one of the most abundant enzymes in the GPx family and is part of the antioxidant defense system developed to maintain ROS. It has been discovered from various sources such as *Thunnus orientalis* (the Pacific bluefin tuna), *Ctenopharyngodon idellus* (the grass carp), *Trematomus bernacchii* (the Antarctic fish) and *Sparus aurata* (the gilthead sea bream) [25].

### 4.2. Manganese peroxidases (MnPs)

Manganese peroxidases (MnPs EC 1.11.1.13) are mainly produced by species such as *B. subtilis*, *Trametes villosa*, *Ceriporiopsis subvermispora*, *Phanerochaete chrysosporium and Phanerochaete tremellosa* [1],[21],[24]. They are heme prosthetic group containing extracellular glycoproteins mainly involved in the removal of lignin from plant cell walls [45],[46]. They catalyze the peroxide-dependent oxidation of Mn (II) to Mn (III) in the presence of Mn (III)-stabilizing ligands resulting in Mn (III) complexes which can then carry out the oxidation of organic substrates [1],[21].

General manganese peroxidase (MnP) mechanism reaction:

$$\text{2 Mn }\left(\text{II}\right)+{\text{2 H}}^{+}+{\text{H}}_{\text{2}}{\text{O}}_{\text{2}}↔\text{2 Mn }\left(\text{III}\right)+{\text{2 H}}_{\text{2}}\text{O}$$

MnPs are lignolytic enzymes that belong to the class II peroxidases, however, their tertiary structure is similar to those of prokaryotic class I peroxidases and they contain disulfide bridges like the class III peroxidases in plants. These disulfide bridges stabilize the MnPs and they are formed as a result of the cysteine amino acid residues they contain. These enzymes merely stable and they are successfully used in various industrial applications such as bioleaching, synthetic dye decolorization, delignification and bio-pulping [6],[21],[35],[46].

### 4.3. Dye-decolorizing peroxidases (DyPs)

**Figure 3.**
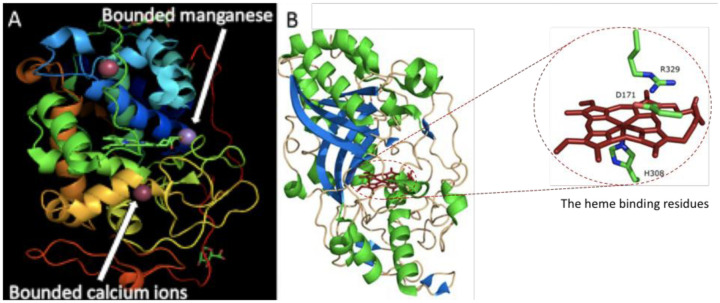
3D structure of (A) manganese peroxidase (MnP) and (B) dye-decolorization peroxidase (DyPs) [8].

Dye-decolorizing peroxidases [DyPs; EC 1.11.1.19] are heme peroxidase enzymes comprising of two domains that contain α-helices and anti-parallel β-sheets (Figure 3) [8]. They are mainly produced by *Bjerkandera adusta* (DyP), *Termitomyces albuminosus* (TAP), *Auricularia auricula-judae* (AjP I), *B. subtilis* (BsDyP), *Marasmius scorodonius* (MsP1 and MsP2), *Exidia glandulosa* (*Egl*DyP), and *Mycena epipterygia* (*Mep*DyP). Even though their physiological functions are unclear, some studies have shown that bacterial DyPs are involved in lignin degradation [8],[10],[18],[26],[47].

These enzymes exhibit a unique substrate acceptance thus, can oxidize substrates that are too large to fit in their active site. They are also active at low pH, which is most likely dictated to the aspartate of the GXXDG motif that functions as an acid-base catalyst at low pH [8],[10]. Yoshida and Sugano [26] observed that their catalytic residue for the reaction with H_2_O_2_ is also different as they use aspartic acid as their catalytic residue instead of the histidine.

DyPs can degrade a variety of dyes, particularly anthraquinone dyes which are poorly accepted by plant and animal peroxidases. They have great potential as biocatalysts in various industrial applications such as dye decontamination and bioremediation due to their ability to oxidize and oxygenate a wide variety of aliphatic and aromatic compounds [8],[26].

With regards to the 3D protein structure (Figures 2 and 3) MnPs is 2 folds higher than dye-decolorizing peroxidase (DyPs) and glutathione peroxidase (GPxs). MnPs has a globular structure containing 11–12 α-helices and thus, stabilized by 10 cysteine amino acid residues which form 5 disulfide bridges. MnPs active site contains a heme cofactor bound by two calcium ions and near its internal heme propionate there are three acidic residues which are used to stabilize Mn (II) or Mn (III) when bound to the enzyme [18],[26].

Other than their distinct tertiary structure, DyPs have a catalytic residue, substrate specificity and optimum pH different to other well-known heme peroxidases. They possess a structure that belongs to the dimeric α + β barrel superfamily in the structural classification of proteins (SCOP) database, whereas other heme peroxidases, such as lignin peroxidase, possess a helix-rich structure [18],[26]. DyPs along with GPxs have a very loosely folded protein structure [18],[26].

## 5. Recent discoveries of peroxidase isoforms from different sources

Due to the wide and increasing biotechnological applications of peroxidases, there is a great need for the discovery of novel and cheaper sources of peroxidases, especially those with better physiochemical parameters than those of known peroxidase isoforms. In the recent years, various peroxidase isoforms have been discovered from different sources through research.

### 5.1. White and red cultivars of Kola nut (Cola nitida) peroxidase isoforms

One of the latest discoveries in peroxidase isoforms took place in Nigeria [16]. The biochemical properties of peroxidases from white and red cultivars of *Cola nitida* were analyzed [16]. *Cola nitida* which belongs to the plant family *Sterculiaceae* and it occupies a unique place among west African countries, where it is widely consumed and used in medical applications, and some religious customs [16]. In their study [16], the presence of three peroxidase isoforms were established in two *C. nitida* species, the white and red Kola nut cultivars.

The Kola nut peroxidases have advantages over horseradish peroxidase and other sources since they were found to be relatively stable in the presence of high H_2_O_2_ concentration [48]. The Kola nut peroxidase retained full activity at 5 mM while horseradish peroxidase lost most of its activity under the same conditions. Hence, it was concluded that the future of the Kola nut peroxidase enzyme in biotechnological applications is quite promising with regards to its improved activity in an organic solvent [48]. According to Adewale and Adekunle [16], the peroxidases obtained from *C. nitidia*, the isoenzymes B of the red *C. nitidia* exhibited the best combination of properties that was found to be useful in biotechnological application.

### 5.2. Sporotrichum thermophile-like catalase-peroxidase gene in Bacillus species

A heme-peroxidase encoding gene was determined through Sanger DNA sequencing and the sequenced gene showed 100% similarity to *Sporotrichum thermophile* catalase-peroxidase gene (*KatG*) in PeroxiBase, as well as deduced protein sequence clustered with bacterial catalase-peroxidase. Catalase-peroxidase (*KatG*) belongs to class I peroxidases of the PCAT superfamily. The corresponding gene (*KatG*) encodes a bifunctional enzyme with predominant catalase and peroxidase activity in an organism [49].

### 5.3. Guaiacol peroxidase in tolerant cocoa (Theobroma cacao L.)

A specific peroxidase isoform was discovered from the cocoa (*Theobroma cacao* L.) hybrids in a study conducted in Cameroon [50]–[52]. Their study focused on the activities and heritability of peroxidases in two hybrid populations of F13 (SNK13 x T79/467) and F79 (T79/467 x SNK13) subjected to *Phytophthora megakarya*. The existence of peroxidase (A2) was discovered after the hybrids were subjected to *P. megakarya*, which is a pathogen causing black pod disease (BOD). This disease is responsible for approximately 30 to 80% of cocoa yield reduction in most African countries [50]–[52].

The existence of a specific peroxidase form (A2) was revealed through the profile of peroxidase isoforms of the mesocarp of infected pods after the infection in tolerant genotypes T79/467, F7902, F7926, F1315 and F1307. These specific isoforms (guaiacol peroxidase-A2) of tolerant hybrid genotypes was found to be related to tolerance, and thus, its existence could be employed in the development of more productive and tolerant varieties [50].

### 5.4. Peroxidases produced by new ligninolytic Bacillus strains.

Various ligninolytic bacteria were isolated from marsh and grassland in Hogsback located in Raymond Mhlaba Municipality, Eastern Cape, South Africa [53]. The isolates were presumed to be ligninolytic due to their ability to utilize alkali lignin as the sole carbon source in an enrichment medium. Ligninolytic organisms have the potential to produce ligninolytic enzymes with significant industrial applications. They are promising candidates for bioremediation, and delignification of feedstock for production of bioethanol. They play a huge role in the valorization of lignocellulosic biomass into other value-added products [53].

The qualitative peroxidase activity of the newly isolated ligninolytic *Bacillus* spp. determined showed peroxidase activity, with the highest potential for peroxidase production exhibited with 6.53 U mL^−1^. The obtained results rendered the isolated ligninolytic *Bacillus* species as promising potentials for peroxidase production [53]. The ligninolytic potential of the isolates was further confirmed by assessing their decolorizing abilities of two ligninolytic indicator dyes, RBBR and CR, with different arene substituent attachment positions. Out of 13 isolates, 5 isolates showed 38.46% decolorization of RBBR while 11 isolated showed up to 84.62% decolorization of CR within 72 hours of incubation [53].

### 5.5. Larimichthys crocea selenium-dependent glutathione peroxidase 1 isoforms

Two selenium-dependent glutathione peroxidase 1 isoforms were identified and characterized for the first-time from *Larimichthys crocea* [25]. Glutathione peroxidases are part of the family of antioxidant enzymes in oxybiotic organisms. They are mainly involved in the anti-pathogen immune response and the antioxidant defense system [25].

Several characteristics such as TGA codon for Sec, SECIS element, active and signature site motifs of GPx family of the two *L. crocea* peroxidase isoforms were revealed through molecular characterization of their nucleotide and their protein sequences [25]. The study concluded that, LcGPx1a and LcGPx1b might play a crucial role in mediating innate immune system response to pathogen attack and could be used as significant biomarkers to detect the impact of environmental factors on fishes.

### 5.6. *Miscanthus x giganteus* peroxidase isoforms

Peroxidase isoforms from *Miscanthus x giganteus* (MxG) were partially characterized and their application for dye degradation was evaluated [34]. The discovery of such peroxidase isoforms from new unexplored sources are of great importance. In the past decade, various peroxidases capable of effectively degrading dyes have been identified from other sources such as white turnip, bitter ground, and horseradish. Accordingly, the newly identified peroxidase isoforms of MxG could be added to the list of effective dye degrading peroxidase sources [11],[34],[54].

### 5.7. Lemon dye decolorizing peroxidase isoforms

In 2016 dye decolorizing abilities were achieved through the discovery of a defense associated peroxidase isoform from young lemon (*Citrus limon*) leaves. The discovery of organic solvent resistant peroxidase is of great importance. The lemon enzyme discovered from *C. limon* was found to exhibit maximum tolerance towards methanol solvent followed by ethanol and isopropanol [11]. With regards to its dye decolorization abilities, the obtained lemon peroxidase was able to effectively decolorize five out of the seven dyes tested. The dyes were quite effectively decolorized in the order: aniline blue, methyl orange, indigo carmine, trypan blue, crystal violet; with congo red and malachite green being not so effectively degraded [11]. It was therefore concluded that the purified lemon peroxidase could be a promising candidate for industrial and analytical applications [11].

This newly discovered peroxidase exhibited stability towards temperature, pH, high concentration of metal salts, heavy metals, organic solvents and it had a tremendous synthetic dye degradation ability which makes it one of the most novel peroxidase isoforms recently discovered [21].

### 5.8. Bacillus subtilis Dye-decolorizing peroxidase (BsDyP)

*Bacillus* strains have been recently isolated as potential lignin-degrading bacteria. In a study conducted in Korea [28], *Bacillus subtilis* KCTC 2023 was chosen as a potential lignin-degrading *Bacillus* strain, and a putative heme-containing dye-decolorizing peroxidase (BsDyP) was discovered in its genome. BsDyP was found to exhibit substrate-dependent optimum temperature. This enzyme showed optimal oxidation activity for high redox potential substrates over +1.0 V versus normal hydrogen electrode (NHE) such as ABTS (2,2′-azino-bis-3-ethylbenzothiazoline-6-sulphonic acid) at 50 °C, but the optimum temperature for decolorizing dyes such as Reactive Black5 and Reactive Blue19 requiring relatively low redox potential (< 1.0V vs. NHE) was determined at 30 °C [28].

The relative activity of the high redox potential substrates at 30°C was also found to be significantly lower than that at 50°C. Thus, BsDyP was identified as bacterial peroxidase with the highest VA oxidation activity among bacterial heme-containing peroxidases previously reported (at the time) and the first to decompose VGE (veratyl glycrol-β-guaiacyl ether) by cleaving C_α_-C_β_ bond independent of oxidative mediators at 50 °C [28]. Accordingly, the unique characteristics of BsDyP discovered in the current study provided insight to great opportunities to realize the microbial pretreatments for producing biofuels and biochemical from lignocellulosic biomass.

### 5.9. Corrosion inducing and inhibiting peroxidases

Microbially influenced corrosion (MIC) is a physical, chemical and microbiological process in which corrosion and electrochemical processes are initiated, facilitated or accelerated by the dynamic support of microorganisms [55]. Microbes derive their energy by oxidizing and reducing a variety of chemical compounds, and they can change their metabolic activities and release various proteins based on the present conditions, of which in some cases lead to MIC.

A study [55] conducted to investigate microorganisms isolated from the corroded carbon steel sample that had been kept in sea water for about 2 years showed the formation of biofilms in all the isolates on carbon steel coupons. The biofilms detached from the coupons were assayed for peroxidase activity. The highest peroxidase were observed from DS4 (± 36U/ml) and the least from DS3 (±19.54 U/ml) with biofilms and almost no peroxidase in the control samples [55]. It was found that peroxidase and catalase were involved in the corrosion and the bacterial strains with the most MIC also produced the highest concentration of these enzymes. Thus, it was concluded that at a certain range these enzymes can induce corrosion whereas higher concentration of both enzymes can inhibit the corrosion. Although the obtained peroxidases were not further studied their ability to induce and inhibit corrosion renders them potential novel peroxidases.

## 6. Applications of peroxidases in industry set up

Peroxidases have the ability to catalyze reaction of a wide range of substrates and they play significant physiological and industrial application roles in nature. The subsequent sections discuss in depth the different roles peroxidase enzymes play.

### 6.1. Rubber degradation

Natural rubber (NR) or *cis*-1,4-poly-isoprene is one of the most important polymers with an estimated annual production of 12.5 million [56]. The use of rubber materials has incredibly increased in the past and after their use, these products are discarded into the environment, thus, environmental pollution [56]. Various efforts have been made to discover rubber degrading microorganisms since 1914, but unfortunately, the biochemical mechanisms of rubber degradation are still unknown [56]. Rubber is very resistant to high temperature and persists in the environment, and it is not easily recycled due to the formation of Sulphur cross-linkages during vulcanization. Thus, the discovery of enzymes and microorganisms capable of biodegrading rubber is of great importance.

A study conducted in Karnataka, India, demonstrated positive results where rubber discs were dumped into the soil to investigate their biodegradation [56]. The dumped discs which were removed from the soil at regular time intervals (2, 4 and 6-months) and plated on the media showed different bacterial isolates belonging to *Pseudomonas* spp. and *Bacillus* spp. [57]. Further investigations showed the isolated *Bacillus pumilus* and the bacterium was able to use NR as its sole carbon source resulting in NR degradation [57] and Laccase and MnP were found to be responsible for the degradation.

### 6.2. Synthetic dye decolorization

Synthetic dyes are broadly used in many industries such as the textile, paper printing, color photography, and additives in petroleum products [6],[13],[56],[58]. Various studies have focused on the decolorization of synthetic dyes by white rot fungi since it can degrade various xenobiotic compounds through the release of enzymes such as LiP, MnP, and laccases. However, it has been proven that enzymatic treatment is simple and more flexible than the use of fungi cultures [60]. Although most of the enzymes used in various industries to decolorize dyes are of fungal origin several bacteria have been used for decolorization of synthetic textile dyes such as *B. cereus* DC11, *B. subtilis*, *Sphingomonas paucimobilis* and *Staphylococcus epidermidis* [20],[41].

Extracellular peroxidase purified from *Bacilllus* sp. F31 was evaluated for its ability to decolorize 16 different industrial dyes, such as basic fuchsin, rhodamine B, methylene blue and malachite green, in a study in 2014 [41]. Out of 16 different textile dyes, the peroxidase was found to efficiently decolorize five dyes of which four were triphenyl methane dyes showing up to 70% decolorization and polymeric heterocyclic dye with approximately 66.2% decolorization. A thermostable peroxidase from *Bacilllus stearothermophilus* has also been found to be stable in decolorization of high-redox potential dyes such as Reactive Blue 5 and Reactive Black 5 [41]. Rendering *Bacillus* spp. F31 and *Bacillus stearothermophilus* peroxidases potential applicant in the textile industry, especially for triphenyl methane dye dominated waste.

Moreover, in a study conducted by Khelil, Choubane [20], *Bacillus* spp. R2 and *B*. *cereus* 11778 were used in single and mixed culture to study the co-production of cellulose and MnP using waste newspaper as sole carbon source. Crude peroxidase produced from these *Bacillus* strains was found to effectively decolorize three synthetic dyes namely methylene blue, neutral red and congo red. Cellulases and peroxidases were rendered as the key enzymes of dye decolorization in this study and the findings were supported by the electrophoresis and zymography profiling of the detected peroxidase isoenzymes [20]. It was therefore concluded that peroxidase enzymes obtained from the two *Bacillus* strains (*Bacillus* spP. R2 and *B. cereus* 11778) could be used in wastewater bioremediation. Thus, *Bacillus* peroxidases have emerged as efficient biological tools for the decolorization of most synthetic dyes in various industries.

### 6.3. Plastic degradation

Approximately 300 million tons of plastic are produced annually worldwide with polyethylene (PE), polypropylene (PP), polyvinyl chloride (PVC), polystyrene (PS), polyurethane (PU) and polyethylene terephthalate (PET) constituting about 80% of the global plastic usage [66]–[68]. The excessive use and the lack of degradability has become of great concern with regards to plastics disposal. Studies have indicated that plastic waste pose a significant threat to domestic animals and marine life, and it contributes to a great deal of environmental pollution [22],[68]. Hence, the need for biodegradable plastics and more effective biodegradation processes has become increasingly important throughout the last decade.

The biological degradation of synthetic plastics is very slow and the extent of degradation is dependent on the structural arrangement of the plastic polymer [22],[68]–[70]. The complete biological degradation is composed of two stages: (i) the breakdown of the polymer into smaller oligomers, and (ii) the eventual cell membrane pass through of monomers which are followed by assimilation and subsequent intracellular metabolism [19],[22],[68]. *Bacillus* spp., *Pseudomonas* spp. and *Arthrobacter* spp. are among the most widely studied degraders of a wide range of plastic polymers through the use of enzymes laccases, lipase, lignin and manganese peroxidase, esterase and amylases [19],[68],[71].

PE can be degraded through molecular mechanisms such as chemical, thermal, photo and biodegradation [22],[67],[72]. With regards to its biodegradation, it has been reported that it can be effectively degraded by several bacterial species through the use of enzymes such as amylase, laccase, LiP and MnP. These enzymes enable the utilization of the PE films by the microorganisms by cleaving their carbon bonds [19],[23],[73]. *Pseudomonas* spp. and *Arthrobacter* spp. isolated from a marine ecosystem are among the species producing enzymes found to be the most effective degraders of high-density PE [68]. Lacasse and MnP from *B. cereus* isolated from local dumpsites in Karnataka, India were identified as key enzymes of PE degradation [67].

PU are characterized by the presence of urethane bonds [68]. They have varied structures such as aromatic-, aliphatic-, polycaprolactone, polyether- and polyester-type PU and they are mostly found in tires, sponges and paints. This type of plastic is insoluble in common solvents such as water, acetone, and ethanol due to the presence of carbamate bonds but despite its durable properties several *Pseudomonas* species such as *P. fluorescens*, *P. aeruginosa* and *P. protegens* along with *B. subtilis* MZA-75 have been identified as some of their most effective degraders [23],[68].

PP is the second wide-spread synthetic worldwide [75]. PP, PVC and polyethylene terephthalate (PET) are among the least reported with regards to biodegradation [67],[68]. These three plastic polymers are highly hydrophobic and resistant to chemical, and they are also thermally and chemically stable hence their biodegradation resistance with or without chemical pretreatment [68]. But despite their stable properties, the biodegradation of these polymers through microbial enzymes have been reported in various studies [73]. Similar results were obtained in Vietnam where three different kinds of plastic bags with different chemical nature were degraded by a novel thermophilic bacterial strain isolated from composting agricultural residual [76].

### 6.4. Wastewater treatment

Removal of phenolic compounds from contaminated water before discharge into any natural stream has become of great importance, as these compounds are toxic to aquatic organisms and humans [77]. Various studies [77] have been conducted to observe the removal of phenol contaminants using peroxidase enzymes, with the aim of developing eco-friendly, economically yet effective biological methods of removing pollutants from wastewater. *Bacillus* spp. has been identified as one of the bacteria capable of peroxidase synthesis and hydrocarbon biodegradation [78]. In an H_2_O_2_ induced sequencing batch reactor (SBR) study, it was discovered that all classes of hydrocarbons could be effectively degraded. Proving that peroxidase-mediated processes is a promising method for efficiently biodegrading concentrated total petroleum hydrocarbons (TPH) laden saline wastewater [78]. Over 99% biodegradation of high concentration of TPH was achieved in the H_2_O_2_-induced SBR operated at a relatively short reaction time using a low dose of H_2_O_2_ [78]. It was therefore concluded that the rate of H_2_O_2_-induced biodegradation of petroleum hydrocarbons is much greater than that of the conventional aerobic biodegradation. The developed process efficiently removed all classes of hydrocarbons without the need to aerate the biomass resulting in prevention of volatilization of light hydrocarbons. It was therefore proven that peroxidase-mediated biodegradation is a simple, low-cost and high rate process for the treatment of hydrocarbon-laden wastewater.

## 7. Conclusions

Discovery of more thermally stable novel peroxidases with better properties such as tolerance to organic solvents, salts, and heavy metals are of paramount importance and further research needs to be explored. Bacteria such as *Bacillus* species from extreme environments are potential sources for various extracellular enzymes with novel properties. Characteristics such as high growth rate, ability to produce extracellular protein in large quantity and general safety is what makes *Bacillus* one of the most important industrial enzyme producers. Many different bacterial strains mostly *Bacillus* spp., due to their constitutive and induced enzymes, have been proven to be involved in the degradation of large and complex compounds into a simpler form. As long as environmental pollution is concerned, excessive use and lack of degradability has become of great concern with regards to plastics disposal. Plastic waste poses a significant threat to human, domestic animals, marine life and contributes to a great deal of environmental pollution. Hence, there is high demand for the discovery of novel thermostable and halotolerant microbial Manganese and Lignin peroxidases besides other peroxidases for effective and efficient biodegradation of plastic wastes and other industrial applications. Furthermore, there is a need to employ non-conventional yet comprehensive tools such as metagenomics approach to mine novel peroxidases of microbial origin with versatile applications from extreme environments.
